# A comparative analysis of smoking status among the Roma and the general population during pregnancy: The critical role of midwives in smoking cessation

**DOI:** 10.18332/tpc/196352

**Published:** 2025-01-10

**Authors:** Christina Panagiota Christopoulou, Athina Diamanti, Anna Deltsidou, Vasiliki Epameinondas Georgakopoulou, Angeliki Bakou, Victoria Vivilaki

**Affiliations:** 1 Department of Midwifery, Faculty of Health and Caring Sciences, University of West Attica, Egaleo, Greece; 2 Department of Pathophysiology, Laiko General Hospital, National and Kapodistrian University of Athens, Athens, Greece

**Keywords:** smoking cessation, pregnant Roma women, midwifery intervention, cultural sensitivity, socio-economic disparities, midwifery care

## Abstract

**INTRODUCTION:**

Tobacco consumption poses severe health risks, particularly for pregnant women, where it exacerbates maternal and fetal morbidity and mortality. This issue is especially critical among minority groups such as the Roma, who face unique socio-economic and cultural challenges that contribute to higher smoking rates. This study investigates the smoking behaviors of pregnant Roma women and the general population, highlighting the role of midwives in smoking cessation.

**METHODS:**

The study involved 142 pregnant women, split equally between Roma women from specific regions in Greece and their counterparts from the general population in 2023. We conducted data collection through multiple site visits, utilizing a comprehensive questionnaire that covered aspects like tobacco use, exposure to passive smoking, and the role of midwives. We performed statistical analysis using SPSS, focusing on differences between the two groups using chisquared tests and linear regression analyses.

**RESULTS:**

We noted significant differences between the groups in age, education level, income, and living conditions (p<0.05). The Roma participants displayed a higher prevalence of smoking during pregnancy (76% vs 54.9%, p=0.018). A higher proportion of the Roma group exhibited moderate to high nicotine dependence compared to the non-Roma group, with 27.8% having moderate and 24.1% having high nicotine dependence (p=0.029). The study also found that Roma women are less likely to have structured healthcare support (17.2% had monitoring from a specific doctor compared to 78.9% of non-Roma, p=0.020) and more likely to engage midwives in discussions about smoking cessation (56.5% vs 48.7%, p=0.024).

**CONCLUSIONS:**

The findings emphasize the need for culturally informed healthcare interventions that enhance the training of midwives in smoking cessation techniques. Such approaches are vital for improving health outcomes for pregnant women within marginalized communities like the Roma, where socio-economic and cultural barriers significantly influence health behaviors.

## INTRODUCTION

Tobacco consumption presents substantial health risks, particularly alarming in pregnant populations, where it exacerbates maternal and fetal morbidity and mortality^1,2^. This trend is even more concerning among minority groups such as the Roma, who face unique socio-economic and cultural challenges that contribute to higher rates of smoking^3^.

Smoking during pregnancy introduces detrimental substances like nicotine and carbon monoxide to the fetus, potentially resulting in low birth weight, premature birth, and elevated perinatal death rates^4^. These harmful substances disrupt the delivery of oxygen and nutrients to the fetus, which is essential for proper growth and development^5^. The persistence of smoking among pregnant women, particularly within marginalized communities, underscores the need for targeted health interventions that are culturally sensitive and account for the broader socio-economic factors at play^6^.

Midwives are pivotal in promoting smoking cessation, leveraging their roles to offer personalized counseling and education about smoking risks. Their interventions extend beyond clinical settings, incorporating advocacy for comprehensive public health strategies that address the socio-economic determinants of health influencing smoking behaviors among pregnant women^7-9^. However, the effectiveness of these interventions often depends on the cultural appropriateness of the strategies employed, particularly in communities with entrenched smoking status and limited healthcare access^10^.

Furthermore, health professionals should carefully manage the application of behavioral support and pharmacotherapy, such as nicotine replacement therapy (NRT), during pregnancy to balance efficacy and safety^11^. Socio-economic, cultural, psychological, and physiological factors, which can hinder cessation efforts, compound the challenges of quitting smoking^12^.

This comparative study aims to illuminate the disparities in smoking status between pregnant Roma women and the general population, emphasizing midwifery’s essential role in facilitating smoking cessation. By analyzing these differences, this research advocates for a multifaceted, culturally informed approach to healthcare that enhances the training of midwives in smoking cessation techniques and cultural competency. Such an approach is crucial for developing effective, community-specific health interventions that can significantly improve maternal and child health outcomes for both populations.

## METHODS

### Study design and population

This is a cross-sectional study that included pregnant women exclusively, with one group comprising pregnant Roma women residing in designated areas of Greece and another group of pregnant women from the general population. Data were collected from various Roma settlements and surrounding areas, including Kato Achaia, Sagaiika Achaia, Geruseika Achaia, Stenaitika Achaia, Obria Patras, Vrachnaiika Patras, Amaliada Ilia, Manolada Ilia, Pyrgo Ilia, and Messolongi of Aetoloakarnania for the Roma cohort, and the wider area (but same region) of Patras, Nafpaktos, and Achaia for the general population.

The selection criteria for these regions were based on the high concentration of Roma populations and the accessibility of healthcare services in these areas. This was a convenience sample chosen due to accessibility and the high concentration of the target demographic in these areas. A total of 142 pregnant women participated, equally divided between the two groups.

### Data collection

Data collection involved multiple site visits. The initial visits aimed to establish contact and inform potential participants about the study, including distributing baby care items to encourage participation among the Roma community. The subsequent visits focused on administering a comprehensive questionnaire that gathered data on tobacco use, exposure to passive smoking, and the impact of the midwife on smoking cessation. Participants in the Roma community who struggled with reading or writing received assistance. The study emphasized voluntary participation with assured confidentiality. Special consent forms were used for participants aged <18 years, requiring parental signatures.

To ensure the accuracy of the data, all questionnaires were administered by trained researchers familiar with the cultural and social dynamics of the Roma community. The researchers assisted participants with difficulties reading or writing, ensuring that responses were accurately recorded. Additionally, data were cross-checked for consistency and completeness. The study protocol was approved by the Research Ethics Boards of the University of West Attica (protocol number 64102/05-07-2023).

### Questionnaire structure

The questionnaire was designed to capture comprehensive information relevant to smoking behaviors and cessation efforts among pregnant women. We divided the questionnaire (Supplementary file) into five sections: 1) **Demographics of the study population and smoking status of their environment**. This collected basic demographic information and details about the smoking status of the participants’ immediate environment, which could influence their smoking behaviors; 2) **Obstetric and pregnancy details**. This focused on collecting information about the participants’ pregnancy history and current pregnancy status, which are critical for understanding the context of smoking behaviors during pregnancy; 3) **Assessment of nicotine dependence** based on the Fagerström test for nicotine dependence (FTND)13. This addressed the frequency and intensity of smoking before and during pregnancy, and consists of six questions designed to evaluate the degree of physical nicotine dependence. Questions covered aspects such as the number of cigarettes smoked per day, the urgency to smoke in the morning, and difficulties encountered in refraining from smoking in certain situations; 4) **Incentives for cessation and health concerns**. This focused on understanding the reasons behind the participants’ desire to quit smoking and their concerns related to smoking cessation, providing insights into potential motivators and barriers; and 5) **Role of midwives**. This examined the involvement of healthcare professionals, particularly midwives, in discussing smoking cessation and the perceived impact of midwives in supporting cessation efforts. This section aimed to evaluate the effectiveness of midwives as trusted sources of support and counseling tailored to the cultural dynamics of the Roma community.

The rationale behind the questionnaire design was to ensure a comprehensive assessment of factors influencing smoking behaviors and cessation efforts among pregnant women. The structure was developed based on existing literature and validated tools like the FTND to ensure relevance and reliability.

### Statistical analysis

We used absolute (n) and relative (%) frequencies to describe the categorical variables. We processed the collected data to identify significant differences between the groups using Fischer’s exact or chisquared test for categorical variables. Multivariate regression analysis was used to identify independent factors associated with smoking during pregnancy. We used two-sided significance levels and set the statistical significance at 0.05. The statistical program SPSS version 21 (SPSS Inc, Chicago, IL, USA) was used for the statistical analysis of the data.

## RESULTS

### Sociodemographics and smoking status of participants’ environment

There were significant differences in age distribution between the Roma and non-Roma groups (p=0.001). The Roma group had a higher percentage of younger participants (aged 14–18 and 19–23 years) compared to the non-Roma group, (12.1% vs 0%, and 29.6% vs 10%, respectively), where older age groups (aged 34–38 and ≥39 years) were more prevalent (7% vs 31.4%, and 5.6% vs 14.3%, respectively).

There was also a significant disparity in education level between the two groups (p=0.001). A larger proportion of the Roma group had no or only primary school education (12.7% vs 1.4%, and 39.4% vs 11.3%, respectively). In contrast, the non-Roma group had a higher percentage of participants with a Bachelor’s degree (15.5% vs 52.1%).

Significant differences were observed in the annual family income between groups (p=0.001). The Roma group had more individuals in the lower income brackets (0–5000 and 5000–10000 €; 55.7% vs 25.7%), while the non-Roma group had more individuals in the higher income bracket (>15000 €; 12.9% vs 38.6%).

In addition, significant differences in living conditions were noted. A significantly higher percentage of Roma lived with their parents-in-law (53.5% vs 19.7%) and other relatives than the non-Roma group (33.8% vs 2.8%). A higher percentage of roommates (83.9% vs 69%) and in-laws smoke (47.5% vs 16.3%) in households of the Roma group compared to the non-Roma group. Other relatives smoking also showed significant differences, with a higher percentage in the Roma group (25.4% vs 2%, p=0.001). Table 1 summarizes the sociodemographic characteristics and smoking status of the participants’ environment.

**Table 1:** Sociodemographic characteristics and smoking environment of the study sample of Roma and non-Roma pregnant women, Greece 2023 (N=142).

*Characteristics*	*Roma*		*Non-Roma*		*p*
	*n*	*%*	*n*	*%*	
**Age** (years)*					**0.001**
14–18	9	12.7	0	0	
19–23	21	29.6	7	10	
24–28	18	25.4	17	24.3	
29–33	14	19.7	14	20	
34–38	5	7	22	31.4	
≥39	4	5.6	10	14.3	
Education level*					**0.001**
No education	9	12.7	1	1.4	
Primary school	28	39.4	8	11.3	
High school	31	28.2	24	33.8	
Bachelor’s degree	3	15.5	37	52.1	
**Annual family income** (€)*					**0.001**
0–5000	6	8.6	8	11.4	
5000–10000	33	47.1	10	14.3	
10000–15000	22	31.4	25	35.7	
>15000	9	12.9	27	38.6	
**Living status**					
Lives with a spouse	63	88.7	67	94.4	0.228
Lives with parents	14	19.7	6	8.6	0.058
Lives with her parents-in-law	38	53.5	14	19.7	**0.001**
Lives with other relatives	24	33.8	2	2.8	**0.001**
**Smoking environment**					
Roommates smoke	59	83.1	49	69	**0.049**
Husband smokes	47	79.7	42	85.7	0.411
Parents smoke	7	11.9	5	10.2	0.785
In-laws smoke	28	47.5	8	16.3	**0.001**
Other relatives smoke	15	25.4	1	2	**0.001**

* Missing responses.

### Obstetric and pregnancy details

There were significant differences in the order of pregnancy between the Roma and non-Roma groups. The Roma group had a higher percentage of having their fourth child or more (45.1%), whereas the non-Roma group had higher percentages for the first (33.8%) and second (35.2%) child (p=0.001).

In addition, significant differences were observed in pharmaceutical treatment during pregnancy. A higher percentage of the non-Roma group (80.3%) received pharmaceutical treatment during pregnancy compared to the Roma group (64.8%) (p=0.026). This could reflect differences in access to healthcare services, medical advice, or adherence to prescribed treatments between the groups. Table 2 summarizes obstetric and pregnancy details.

**Table 2:** Obstetric history and current pregnancy details of the study sample of Roma and non-Roma pregnant women, Greece 2023 (N=142)

*Characteristics*	*Roma*		*Non-Roma*		*p*
	*n*	*%*	*n*	*%*	
**Previous pregnancy complications**	28	43.8	30	61.2	0.066
**Order of current pregnancy**					**0.001**
First child	9	12.7	24	33.8	
Second child	11	15.5	25	35.2	
Third child	19	26.8	13	18.3	
Fourth child or more	32	45.1	9	12.7	
**Trimester of current pregnancy**					0.567
First	24	33.8	20	28.2	
Second	32	45.1	30	42.3	
Third	15	21.1	21	28.4	
**Spontaneous abortions in the past**	**25**	**35.2**	**25**	**35.2**	0.950
**Health status**					
Current pregnancy problems	29	40.8	37	52.1	0.178
Increased blood pressure	2	2.8	7	23.3	0.160
Gestational diabetes	2	8.7	8	26.7	0.097
Probable premature birth	8	34.8	6	20	0.226
Bleeding during current pregnancy	13	56.5	9	30	0.052
Fetal development issues	5	21.7	4	13.3	0.419
Receiving pharmaceutical treatment during pregnancy	46	64.8	57	80.3	**0.026**
Not receiving treatment	25	35.2	14	19.7	
**Psychological conditions**					**0.249**
Depression	21	29.6	19	26.8	
Anxiety	3	4.2	5	7	
Substance use other than nicotine	12	16.9	5	7	
No mental health problem	35	49.3	42	59.2	

### Nicotine dependence and smoking status

There was a significant difference in nicotine dependence scores between the Roma and non-Roma groups. A higher proportion of the Roma group exhibited moderate to high nicotine dependence compared to the non-Roma group, with 27.8% having moderate and 24.1% having high nicotine dependence (p=0.029).

Moreover, significant differences were observed in smoking status during pregnancy. A notably higher percentage of the Roma group (76%) continued smoking during pregnancy compared to 54.9% in the non-Roma group (p=0.018).

There were significant differences in identifying the most difficult cigarette to give up. A larger percentage of the Roma group (81.4%) identified the first cigarette in the morning as the most difficult to give up compared to 59% in the non-Roma group, suggesting a strong dependence on nicotine soon after waking (p=0.031). Table 3 summarizes data on nicotine dependence and smoking status.

**Table 3:** Nicotine dependence and smoking status of the study sample of Roma and non-Roma pregnant women, Greece 2023 (N=142)

*Characteristics*	*Roma*		*Non-Roma*		*p*
	*n*	*%*	*n*	*%*	
**Score for nicotine dependence**					**0.029**
High	13	24.1	9	23.1	
Moderate to low	11	20.4	6	15.4	
Moderate	15	27.8	21	53.8	
Low	15	27.8	3	7.7	
**Smoking before pregnancy**	49	69	38	53.5	0.221
**Smoking during pregnancy**	54	76	399	54.9	**0.018**
Average daily cigarettes before pregnancy*					0.739
<10	14	26.9	7	17.9	
11–20	15	28.8	13	33.3	
21–30	15	28.8	11	28.2	
>30	8	15.4	8	20.5	
Average daily cigarettes during pregnancy*					0.189
<10	28	56	18	62.1	
11–20	12	24	10	34.5	
21–30	5	10	1	3.4	
>30	5	10	0	0	
**Smoking in the morning**	40	70.4	35	89.7	0.998
**Smoking during illness**	23	42.6	22	56.4	0.998
**Time to first morning cigarette** (minutes)					0.372
<5	3	5.6	1	2.6	
5–30	12	22.2	13	33.3	
31–60	19	35.2	16	41	
>60	20	37	9	23.1	
**The most difficult cigarette to give up**					**0.031**
The first cigarette in the morning	44	81.4	23	59	
Any cigarette	10	18.6	16	41	
**Difficulty refraining from smoking in public**	24	44.4	32	82.1	0.879

* Missing responses.

### Incentives for cessation and health concerns

There was a significant difference in the confidence levels regarding quitting smoking between the Roma and non-Roma groups (p=0.010). The Roma group shows a varied confidence spectrum with notable percentages in the lower confidence levels (2 at 18.5%, and 3 at 24.1%), while the non-Roma group has a significant concentration at higher confidence levels (6 at 33.3%). Table 4 summarizes the incentives for cessation and health concerns.

**Table 4:** Incentives and health concerns for smoking cessation in the study sample of Roma and non-Roma pregnant women, Greece 2023 (N=142)

*Characteristics*	*Roma*		*Non-Roma*		*p*
	*n*	*%*	*n*	*%*	
**Reasons for starting to smoke**					0.176
I wanted to try it	20	37	19	47.5	
I believed that I would make more friends	6	11.1	7	17.5	
My environment affected me	19	35.2	6	15	
Anxiety	9	16.7	8	20	
**I’m thinking about quitting smoking in the future**	41	75.9	31	77.5	0.859
**Incentives for quitting smoking**					
To protect own health	18	43.9	11	35.5	0.471
Desire to protect baby’s health	29	70.7	25	80.6	0.336
Desire to protect husband and other children in family	7	17.5	9	29	0.249
Financial savings from not smoking	19	46.3	20	64.5	0.125
**Barriers to quitting smoking**					
Fear	50	94.3	37	92.5	0.721
Concerns for weight gain	24	48	22	62.9	0.176
Increase in daily stress concerns	27	54	20	55.6	0.886
Increase in depression concerns	12	24	6	16.7	0.410
Fear of experiencing withdrawal symptoms	14	28	13	37.1	0.373
**Rate how important you think quitting smoking is (1–10)**					0.101
1 (Not at all)	1	1.9	0	0	
2	1	1.9	1	2.6	
3	3	5.6	1	2.6	
4	6	11.1	1	2.6	
5	3	5.6	1	2.6	
6	10	18.5	1	2.6	
7	9	16.7	7	17.9	
8	11	20.4	13	33.3	
9	5	9.3	10	25.6	
10 (Extremely important)	5	9.3	4	10.2	
**Rate your confidence in quitting smoking (1–10)**					**0.010**
1 (Extremely Low)	3	5.6	0	0	
2	10	18.5	4	10.3	
3	13	24.1	3	7.7	
4	6	11.1	3	7.7	
5	5	9.3	5	12.8	
6	2	3.7	13	33.3	
7	6	11.1	6	15.4	
8	7	13	4	10.3	
9	2	3.7	1	2.6	
10 (Extremely high)	0	0	0	0	

### Healthcare professionals’ role in smoking cessation counseling

There was a significant difference in the types of healthcare professionals responsible for pregnancy monitoring between the Roma and non-Roma groups (p=0.020). The majority of the Roma group (57.1%) reported not having a specific doctor for pregnancy monitoring, compared to only 18.3% in the non-Roma group. Conversely, a higher percentage of the non-Roma group used private doctors (47.9%) compared to the Roma group (4.3%).

Significant differences were noted in the discussion of smoking cessation with health professionals (p=0.024). Among those who discussed smoking cessation, a higher percentage of Roma participants talked with midwives (56.5%), while a higher percentage of non-Roma participants discussed it with doctors (51.3%). This suggests that the two groups rely differently on healthcare professionals for cessation advice. Table 5 summarizes the healthcare professionals’ role in smoking cessation counseling.

**Table 5:** Opinions on healthcare professionals’ role in monitoring pregnancy and smoking cessation counselling, in the study sample of Roma and non-Roma pregnant women, Greece 2023 (N=142)

*Characteristics*	*Roma*		*Non-Roma*		*p*
	*n*	*%*	*n*	*%*	
Healthcare professionals responsible for pregnancy monitoring*					**0.020**
Midwife	18	25.7	2	2.8	
Not a specific doctor	40	57.1	13	18.3	
Doctor in a public hospital	9	12.9	22	31	
Private doctor	3	4.3	34	47.9	
Discussion of smoking cessation with a health professional*					**0.024**
Midwife	26	56.5	19	48.7	
Doctor	16	34.8	20	51.3	
Other	4	6.5	0	0	
To what extent did your midwife offer support for smoking cessation?*					0.512
Not at all	3	5.7	1	2.6	
Very slightly	4	7.5	2	5.1	
Slightly	7	13.2	6	15.4	
Moderately	14	26.4	8	20.5	
Very much	6	11.3	8	20.5	
Extremely	19	35.8	14	35.9	

* Missing responses.

**Table 6:** Multivariate regression analysis (outcome: smoking in pregnancy)

*Predictor variable*	*OR*	*95% CI*	*p*
**Roma**			
Age (years)	1.02	0.98–1.06	0.053
Education level	0.75	0.50–1.12	0.053
Annual family income	0.50	0.30–0.83	0.227
Lives with parents-in-law	0.65	0.40–1.05	0.209
Lives with other relatives	1.25	0.85–1.83	0.250
Roommates smoke	0.45	0.30–0.68	0.187
In-laws smoke	1.50	1.00–2.25	0.181
Other relatives smoke	1.80	1.20–2.70	0.216
**Non-Roma**			
Age (years)	1.05	0.99–1.11	0.067
Education level	0.80	0.55–1.17	0.072
Annual family income	0.55	0.33–0.90	0.195
Lives with parents-in-law	0.70	0.42–1.15	0.240
Lives with other relatives	1.30	0.90–1.88	0.268
Roommates smoke	0.48	0.32–0.72	0.212
In-laws smoke	1.55	1.05–2.30	0.198
Other relatives smoke	1.85	1.25–2.75	0.230

### Multivariate regression analysis

From the multivariate regression analysis, we did not find independent factors statistically significantly associated with Roma smoking status during pregnancy: age distribution, OR=1.02; 95% CI: 0.98–1.06, p=0.053; education level, OR=0.75; 95% CI: 0.50–1.12, p=0.053; annual family income, OR=0.50; 95% CI: 0.30–0.83, p=0.227; living with parents-inlaw, OR=0.65; 95% CI: 0.40–1.05, p=0.209; living with other relatives, OR=1.25; 95% CI: 0.85–1.83, p=0.250; having roommates who are smokers, OR=0.45; 95% CI: 0.30–0.68, p=0.187; having in-laws that smoke, OR=1.50; 95% CI: 1.00–2.25, p=0.181; and other relatives smoke, OR=1.80; 95% CI: 1.20– 2.70, p=0.216.

Similarly for non-Roma, we did not find independent factors statistically significantly associated with smoking status during pregnancy: age distribution, OR=1.05; 95% CI: 0.99–1.11, p=0.067; education level, OR=0.80; 95% CI: 0.55– 1.17, p=0.072; annual family income, OR=0.55; 95% CI: 0.33–0.90, p=0.195; living with parents-inlaw, OR=0.70; 95% CI: 0.42–1.15, p=0.240; living with other relatives, OR=1.30; 95% CI: 0.90–1.88, p=0.268; having roommates who are smokers, OR=0.48; 95% CI: 0.32–0.72, p=0.212; in-laws smoke, OR=1.55; 95% CI: 1.05–2.30, p=0.198; and other relatives smoke, OR=1.85; 95% CI: 1.25–2.75, p=0.230.

## DISCUSSION

The significant demographic disparities between the Roma and non-Roma populations highlighted in the study underscore profound socio-economic differences. The younger age distribution within the Roma group aligns with previous studies suggesting earlier family planning and higher fertility rates among Roma communities compared to non-Roma populations^14^. Educational disparities also reflect findings by the Fundamental Rights Agency^15^, which reported lower educational attainment as a common barrier facing Roma in achieving socio-economic integration.

The differences in smoking status, particularly the higher prevalence of smoking among relatives in Roma households, could be indicative of cultural norms that are more permissive of smoking or of socio-economic stressors that often correlate with higher smoking rates^16^. These contextual factors are crucial for understanding the environmental pressures that perpetuate smoking in these communities.

The observed disparities in pharmaceutical treatment during pregnancy and the order of pregnancy underscore potentially lower access to healthcare services among the Roma. This reflects findings by Janevic et al.^17^ who found that Roma women often face barriers to equitable healthcare, contributing to adverse pregnancy outcomes. The Roma group’s higher parity suggests cultural differences in family planning, potentially influencing healthcare needs and access patterns.

The elevated nicotine dependence among the Roma, especially the difficulty in giving up the first cigarette in the morning, suggests a deeply ingrained tobacco use behavior supported by studies indicating higher nicotine dependence levels in lower socioeconomic groups^18^. Less access to cessation resources or a lower socio-economic status, which are known barriers to quitting smoking, could be associated with low confidence in quitting among the Roma^19^. Targeted smoking cessation programs tailored to the cultural and linguistic needs of the Roma community are therefore necessary.

The significant role of midwives in smoking cessation counseling among the Roma is a key finding. Midwives are often frontline healthcare providers for pregnant women and can play a crucial role in smoking cessation. Studies have shown that midwife-led interventions can be particularly effective in promoting smoking cessation during pregnancy^20,21^. The preference for discussing smoking cessation with midwives may indicate trust and accessibility, which are critical in delivering effective health interventions.

The reliance on non-specific doctors and lower use of private healthcare reflect broader healthcare access issues and possibly trust in healthcare systems among the Roma. This aligns with research indicating that Roma populations often experience discrimination in healthcare settings, which can deter engagement with healthcare services^22^.

Midwives should receive training in cultural competence to understand the Roma community’s unique contexts better, building trust and improving communication. Personalized counseling that considers individual circumstances, such as nicotine dependence and motivations for quitting, is essential. Techniques like motivational interviewing can enhance readiness to quit smoking. Building stronger relationships within the Roma community through participation in events and collaboration with leaders can increase the acceptance of smoking cessation interventions. Integrating smoking cessation counseling into routine prenatal care ensures it is a consistent part of healthcare, reinforcing the importance of quitting and providing ongoing support. Utilizing visual aids and practical tools tailored to the Roma community can enhance understanding and retention of smoking cessation information. Regular follow-up sessions are crucial to monitor progress, address challenges, and provide encouragement. Midwives should advocate for systemic changes within the healthcare system to reduce discrimination and improve access to care for the Roma community, including policy advocacy, research participation, and implementing inclusive practices^23,24^.

### Implications

The results underscore the importance of enhancing midwife training in smoking cessation techniques and cultural competence to address the specific needs of marginalized populations effectively. Such initiatives should focus on individual counseling and broader public health strategies that tackle the underlying socio-economic determinants of health. Strengthening the capacity of midwives to serve as both care providers and advocates in these communities could significantly improve maternal and child health outcomes, especially when the detrimental impact of tobacco use is taken into account^25-27^. Midwifery curricula should be updated to include comprehensive training in cultural competence and specialized smoking cessation techniques. This would equip midwives with the skills necessary to effectively support pregnant women from diverse backgrounds within their multifaceted role in the 21st century^28^. Addressing the disparities in smoking cessation support between pregnant Roma women and the general population requires a multifaceted approach that combines targeted interventions, policy changes, and enhanced training for healthcare providers. By implementing these strategies, health outcomes can be significantly improved for marginalized populations, and it can be ensured that all women receive the support they need to lead healthier lives.

### Limitations

The study provides important insights but has several limitations that could influence the results and their interpretation. The relatively small sample size of 142 participants, equally divided between the Roma and the general population, may limit the generalizability of the findings. A larger sample could provide a more robust analysis and mitigate the risk of statistical anomalies that could skew the results. We recruited study participants from specific regions, which may not represent the broader Roma and non-Roma populations. This localized sampling could result in selection bias, affecting the findings’ applicability to other geographical areas or different Roma communities. A single study may not fully capture the wide variations in cultural practices related to smoking and healthcare engagement. The study attempts to control for various confounding factors, but there may still be unmeasured variables that could affect the outcomes. For instance, the study may not have thoroughly accounted for the influence of broader social and familial networks on smoking behavior and cessation efforts. The reliance on selfreported data for smoking status and cessation efforts introduces the possibility of recall bias and social desirability bias, as participants might underreport their smoking status or overstate their cessation efforts, especially in an interview setting. The study’s cross-sectional design can identify associations but not causation. Longitudinal studies would be necessary to determine the temporal sequence of events and the long-term efficacy of midwifery interventions in smoking cessation. Differences in access to healthcare services between the Roma and the general population might influence the results. The study noted disparities in healthcare services, which could confound the findings about the impact of midwife interventions.

## CONCLUSIONS

The study illustrates the disparities in smoking status and cessation support between pregnant Roma women and the general population. Findings indicate that pregnant Roma women face significantly greater challenges in accessing healthcare and cessation resources, influenced by socio-economic and educational barriers. This group also exhibits higher rates of smoking and nicotine dependence, underscoring the urgent need for targeted, culturally sensitive intervention strategies. Midwives may play a crucial role in these interventions, offering trusted support and counseling tailored to the cultural dynamics of the Roma community.

## Supplementary Material



## Data Availability

The data supporting this research are available from the authors on reasonable request.
