# Supplementary material for: A comparative analysis of smoking status among the Roma and the general population during pregnancy: The critical role of midwives in smoking cessation
Source: Tob Prev Cessat. 2025 Jan 10;11:10.18332/tpc/196352. doi: 10.18332/tpc/196352 (PMC11719057; doi:10.18332/tpc/196352)
Supplement: Supplementary file 1 [file TPC-11-03-s1.pdf]

Questionnaire

SECTION 1

Demographics of the study population and smoking habits of their environment.

Please choose the correct answer.

**At which of the following age groups do you belong to?**
a. 14-18 years
b. 19-23 years
c. 24-28 years
d. 29-33 years
e. 34-38 years
f. ≥39 years
**What is your highest educational level?**
a. No education
b. Primary school
c. High school
d. Bachelor's degree
**What is your annual family income?**
a. 0-5,000 euros
b. 5,000-10,000 euros
c. 10,000-15,000 euros
d. Over 15,000 euros
**Living conditions**
**Do you live with a spouse?**
a. Yes
b. No
**Do you live with your parents?**
a. Yes
b. No
**Do you live with your parents-in-law?**
a. Yes
b. No
**Do you live with other relatives?**
a. Yes
b. No

**Smoking habits in the household**
**Do your roommates smoke?**
a. Yes
b. No
**Does your husband smoke?**
a. Yes
b. No
**Do you parents smoke?**
a. Yes
b. No
**Do your parents in-law smoke?**
a. Yes
b. No
**Do other relatives smoke?**
a. Yes
b. No

SECTION 2

Obstetric and pregnancy details

Please choose the correct answer.

**Did you have complications in your previous pregnancy?**
a. Yes
b. No
**Order of current pregnancy**
a. First Child
b. Second Child
c. Third Child
d. Fourth Child or More
**Trimester of current pregnancy**
a. First Trimester
b. Second Trimester
c. Third Trimester
**Di you have spontaneous abortions in the past?**
a. Yes
b. No

**Do you experience problems during the current pregnancy?**
a. Yes
b. No
**Do you suffer from increased blood pressure?**
a. Yes
b. No
**Do you suffer from gestational diabetes?**
a. Yes
b. No
**Is there a possibility for premature birth?**
a. Yes
b. No
**Did you have bleeding during current pregnancy?**
a. Yes
b. No
**Are there any fetal development issues?**
a. Yes
b. No
**Are you receiving any pharmaceutical treatment during this pregnancy?**
a. Yes
b. No
**Do you suffer from any of the following psychological conditions?**
a. Depression
b. Anxiety
c. Substance Use Other Than Nicotine
d. No mental health problem

SECTION 3

Nicotine Dependence and smoking habits: Use of the Fagerström questionnaire and details about the smoking habits before the current pregnancy

Please choose the correct answer.

**Are you smoking during the current pregnancy?**
a. Yes
b. No
**How soon after you wake up do you smoke your first cigarette?**

a. <5 min
b. 5-30 min
c. 31-60 min
d. >60 min
**Do you find it difficult to refrain from smoking in the places where it is forbidden (e.g., in church, at the library, in cinema)?**
a. Yes
b. No
**Which cigarette would you hate most to give up?**
a. The first cigarette in the morning
b. Any cigarette
**How many cigarettes/day do you smoke?**
a.<10
b. 11-20
c. 21-30
d. >30
**Do you smoke more frequently during the first hours after waking than during the rest of the day?**
a. Yes
b. No
**Do you smoke if you are so ill that you are in bed most of the day?**
a. Yes
b. No
**Did you smoke before the current pregnancy?**
a. Yes
b. No
**How many cigarettes/day did you smoke before the current pregnancy?**
a. <10
b. 11-20
c. 21-30
d. >30

SECTION 4

Incentives for cessation and health concerns

**Which was the main reason for starting smoking?**
a. I wanted to try it.
b. I believed that I would make more friends.

c. My environment affected me.
d. Anxiety
**Are you thinking about quitting smoking in the future?**
a. Yes
b. No
**Which is the main incentive for quitting smoking?**
a. To protect her own health
b. Desire to protect the baby's health
c. Desire to protect my husband and other children
d. Financial savings from not smoking
**Which is the main barrier to quitting smoking?**
a. Fear
b. Concerns for weight gain
c. Increase in daily stress concerns
d. Increase in depression concerns
e. Fear of experiencing withdrawal symptoms
**Rate how important you think quitting smoking is (1–10).**
a. 1
b. 2
c. 3
d. 4
e. 5
f. 6
g. 7
h.8
i. 9
j.10
**Rate your confidence in quitting smoking (1–10).**
a. 1
b. 2
c. 3
d. 4
e. 5
f. 6
g. 7

h.8
i. 9
j.10

SECTION 5

Healthcare professionals' role

Please choose the correct answer.

**Healthcare professionals responsible for pregnancy monitoring (You can choose more than one answer)**
a. Midwife
b. Not a specific doctor
c. Doctor in a public hospital
d. Private doctor
**Discussion of smoking cessation with a health professional (You can choose more than one answer)**
a. Midwife
b. Doctor
c.Other
**To what extent did your midwife offer support for smoking cessation?**
a. Not at all
b. Very slightly
c. Slightly
d. Moderately
e. Very much
f. Extremely

© 2025 Christopoulou C.P. et al.
